# User Compliance With the Health Emergency and Disaster Management System: Systematic Literature Review

**DOI:** 10.2196/41168

**Published:** 2023-05-05

**Authors:** Widia Resti Fitriani, Juliana Sutanto, Putu Wuri Handayani, Achmad Nizar Hidayanto

**Affiliations:** 1 Faculty of Computer Science Universitas Indonesia Depok Indonesia; 2 Department Human Centred Computing, Faculty of Information Technology Monash University Melbourne Australia

**Keywords:** health emergency and disaster management system, systematic literature review, user compliance, theoretical lens, influential factors, contact-tracing application

## Abstract

**Background:**

Health-related hazards have a detrimental impact on society. The health emergency and disaster management system (Health EDMS), such as a contact-tracing application, is used to respond to and cope with health-related hazards. User compliance with Health EDMS warnings is key to its success. However, it was reported that user compliance with such a system remains low.

**Objective:**

Through a systematic literature review, this study aims to identify the theories and corresponding factors that explain user compliance with the warning message provided by Health EDMS.

**Methods:**

The systematic literature review was conducted using Preferred Reporting Items for Systematic reviews and Meta-Analyses 2020 guidelines. The search was performed using the online databases Scopus, ScienceDirect, ProQuest, IEEE, and PubMed, for English journal papers published between January 2000 and February 2022.

**Results:**

A total of 14 papers were selected for the review based on our inclusion and exclusion criteria. Previous research adopted 6 theories when examining user compliance, and central to the research was Health EDMS. To better understand Health EDMS, based on the literature reviewed, we mapped the activities and features of Health EDMS with the key stakeholders involved. We identified features that require involvement from individual users, which are surveillance and monitoring features and medical care and logistic assistance features. We then proposed a framework showing the individual, technological, and social influencing factors of the use of these features, which in turn affects compliance with the warning message from Health EDMS.

**Conclusions:**

Research on the Health EDMS topic increased rapidly in 2021 due to the COVID-19 pandemic. An in-depth understanding of Health EDMS and user compliance before designing the system is essential for governments and developers to increase the effectiveness of Health EDMS. Through a systematic literature review, this study proposed a research framework and identified research gaps for future research on this topic.

## Introduction

### Background

Emergencies and disasters threaten the safety of human life and trigger acute feelings of stress, anxiety, and uncertainty [1]. The increasing number of disasters and the exposure of people and property to hazards have prompted an increased interest in and support for emergency and disaster management (EDM) policies and programs [2]. The terms “emergency management” and “disaster management” are often used interchangeably. Disaster management is the organization, planning, and application of measures to prepare for, respond to, and recover from disasters [3,4]. Meanwhile, emergency management is defined as a strategic management process to protect critical assets from hazards, save lives, minimize property or environmental damage, and reduce suffering [5,6]. During disaster or emergency management, there is a need to coordinate the efforts within and across organizations and securely exchange data and share information through a system [7].

Previous studies [8-11] have distinguished between the emergency management system (EMS) and the disaster management system (DMS). An EMS aims to handle emergency planning, control, and reduction with 3 essential components: hazard detection, emergency management, and public communication [8,9]. Meanwhile, a DMS consists of 3 application domains: monitoring, response, and forecasting the expected disasters [10]. In addition, a DMS includes the functionality of information sharing, search and rescue missions, and damage assessment [11]. In general, the functionality of the EMS and DMS follows the EDM phases, which are mitigation, preparedness, response, and recovery. This study uses the term “emergency and disaster management system” (EDMS) to cover all the EDM phases.

With the increasing risk of emergency and disaster, research related to EDMSs is constantly evolving for various hazard types, including health-related hazards. Health-related hazards, such as disease outbreaks (eg, COVID-19, avian influenza, and Ebola), are 1 of the most common hazardous events [9]. From 2012 to 2017, the World Health Organization (WHO) recorded more than 1200 outbreaks in 168 countries, including those due to new or re-emerging infectious diseases. In 2018, another 352 infectious disease events emerged. Several disease outbreaks are classified as Public Health Emergency for International Concern (PHEIC) by WHO, including the 2009 H1N1 influenza pandemic, Ebola (2013-2015 West African outbreak and 2018-2020 outbreak in the Democratic Republic of Congo), poliomyelitis (2014 to present), Zika (2016), and COVID-19 (2020 to now) [7]. From the ongoing COVID-19 pandemic, there have been more than 349 million cases of COVID-19 worldwide, including more than 5.5 million deaths as of January 31, 2022 [12]. Moreover, the International Monetary Fund estimates that the global economy shrunk by 4.4% in 2020 compared to 2019 due to the COVID-19 pandemic [13].

Health-related hazards have different characteristics from natural disasters and disasters caused by humans. When a natural disaster or human-induced disaster occurs, people are advised to evacuate to a safe place. In contrast, during a pandemic, people must stay at home or be quarantined to prevent disease transmission [14]. As infectious diseases spread quickly, they require fast and appropriate treatment [14]. Various media and information sources, such as mass media, text messages, social media, and notifications in the community, can be used to disseminate warnings [15]. During the COVID-19 pandemic, self-administered warning systems or applications, called contact-tracing applications (CTAs), have been widely used to disseminate alerts and recommendations [16,17]. A CTA is a form of the health emergency and disaster management system (Health EDMS) that is used to prevent, prepare for, respond to, and deal with emergencies and disasters that threaten public health [3]. Health EDMS can be applied to all stages of EDM, but the response stage requires user involvement the most. During the response phase, Health EDMS performs disease monitoring and tracing, provides medical care, and disseminates information and warnings [18-21]. Risk communication plays a vital role in attracting user attention and encouraging compliance to Health EDMS warnings.

Understanding individual behavior toward Health EDMS is essential because health EDM is not solely the government's responsibility [2]. Individuals are an integral part of the system as they are responsible for the health of themselves, their families, and their neighbors [2]. Previous studies [22-30] have identified the factors driving individuals' adoption of Health EDMS. In addition to these studies, 2 literature reviews [31,32] have summarized the determining factors of individuals' acceptance and adoption of Health EDMS. The first literature review compiled 25 studies of individuals' acceptance and adoption of a CTA. The second literature review conducted a systematic review of 21 national COVID-19 CTAs and verified CTA quality and public adoption [32]. These reviews have shown that engagement could increase adoption and that there is an association between higher application adoption and lower infection rates [32]. Several other literatures reviewed on the Health EDMS topic have discussed the privacy concerns [33-35] and the digital solutions for dealing with the COVID-19 outbreak [36,37].

The extant literature reviews focus on the acceptance and adoption of Health EDMS. Although they have offered valuable insights into the development of studies in the Health EDMS field, little attention has been paid to how effective the warning message provided by Health EDMS generates user compliance [38]. According to Han et al [38], Abdelhamid et al [39], and O’Malley et al [40], compliance with warning messages sent by Health EDMS is crucial to determining the EDMS's effectiveness. Users who comply with Health EDMS's warning message play an active role in preventing disease transmission [38]. However, the compliance rate is reported to be low in the context of health-related hazards [27,41]. Therefore, it is important to understand the factors affecting user compliance with Health EDMS's warning message. This paper addresses the following question: What theories and corresponding factors explain user compliance with the warning message provided by Health EDMS?

To answer this question, we reviewed concepts related to hazards, the EDM cycle, and EDMS features from previous research to understand the activities and stakeholders involved in EDM. After that, we mapped the EDMS feature into a CTA to find out which EDMS features have been implemented to deal with health-related hazards. We also analyzed the roles and authorization of stakeholders in each feature to find out which features involve user participation and compliance. Through this mapping, this research can make a specific contribution regarding user participation in Health EDMS and compliance with Health EDMS’s warning message.

Our systematic review followed the Preferred Reporting Items for Systematic Reviews and Meta-Analyses (PRISMA) 2020 method because it has clear, structured, transparent, and complete reporting of systematic reviews [42]. The target audience for this review was threefold: first, researchers who are interested in pursuing research on compliance with the warning message provided by Health EDMS; second, organizations and governments that need information about what related factors can influence the design and implementation of Health EDMS; and third, Health EDMS vendors or developers who would like to understand the sociotechnical factors affecting the compliance of warning messages prior to Health EDMS implementation.

This paper is organized into 5 sections. Section 1 describes the research background and explains the key concepts, and the research methodology is discussed in Section 2. Next, the results and discussion of this study are elaborated in Sections 3 and 4. The final section concludes this study.

### Key Concepts

#### Hazard

Before discussing emergencies and disasters, it is important to understand the definition and classification of hazards. A hazard is a process, phenomenon, or human activity that can harm people's lives and health, damage property, disrupt social and economic activities, and damage the environment [3]. The WHO classification of hazards generally consists of natural, human-induced, and environmental hazards, as provided in Multimedia Appendix 1 [36].

Hazards have the potential to create any scale of emergency or disaster. Emergency and disaster, at first glance, have a similar meaning, but there are fundamental differences between the two. An emergency can be defined as a severe disruption to the functioning of a community or society, causing human, material, economic, or environmental impacts, which can be overcome by the internal resources of the community and society itself [43]. However, a disaster is a severe disturbance that impacts communities and society and cannot be overcome solely by relying on internal resources [43]. In other words, a disaster is an event that causes significant damage or loss and thus requires resources beyond a community's capability and multiple agency responses [44]. Thus, an event is declared a disaster if there is a need for external assistance to address its impact [45]. Both emergencies and disasters must be managed to prevent or minimize their impacts.

#### Emergency and Disaster Management

**Figure 1:**
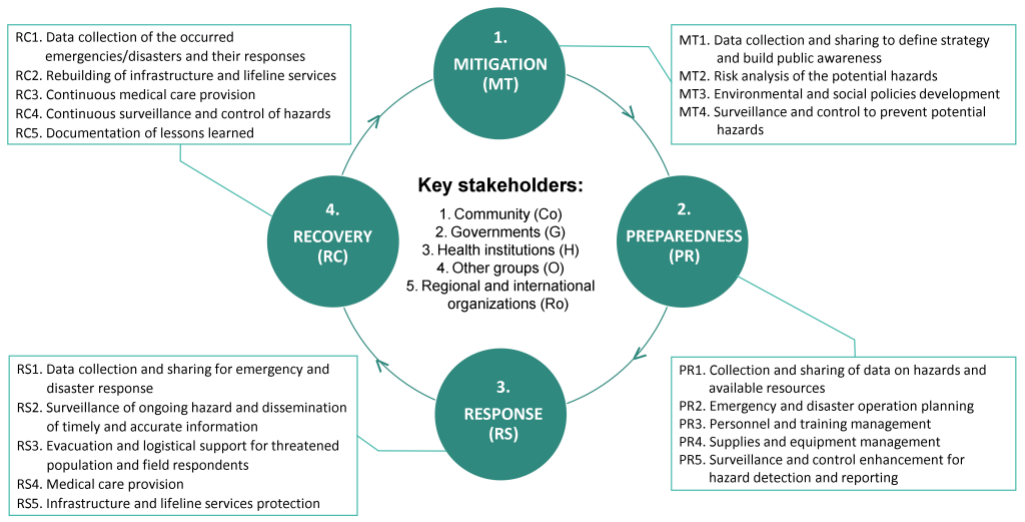
The EDM cycle. EDM: emergency and disaster management.

To answer the research question, we need to understand the general concept of EDM. As previously mentioned, the terms “emergency management” and “disaster management” are often used interchangeably because they have considerable overlap [3]. The EDM cycle consists of 4 phases, namely mitigation, preparedness, response, and recovery [11,45,46], as shown in Figure 1. The mitigation and preparedness phases are part of risk assessments before a hazardous event [45]. Mitigation is the application of actions to prevent a hazard from occurring or reduce its impact [18]. Although the impact of a hazard is often not wholly preventable, various strategies and actions can substantially reduce its scale or severity. Activities during the mitigation phase include engineering techniques and hazard-resistant construction, environmental and social policy development, and public awareness enhancement [3].

The second phase, preparedness, aims to prepare the community to respond to a hazard [18] by providing information, creating partnerships, developing plans, building resources, and creating procedures [47]. When a hazardous event occurs, response and recovery must be carried out [45]. The response actions directly before, during, or immediately after a hazardous event occurs can save lives, reduce health impacts, ensure public safety, and meet the basic needs of the affected people [3]. Activities during the response phase include evacuation, search and rescue, medical care, and temporary housing [3,18]. The response activities may also extend to the recovery stage [45]. Recovery is a long-term action taken after the immediate effects of a hazardous event have passed in order to stabilize society and return to normalcy [18]. Recovery also aims to restore or improve the livelihoods and health of the affected populations and restore the economic, physical, social, cultural, and environmental systems [3].

Each activity shown in Figure 1 requires cooperation and coordination among the various parties, including the community, governments, health institutions, other groups, and regional and international organizations [48]. The community includes at-risk populations, survivors, and community groups or organizations [48]. Governments at all levels include leaders and politicians, ministries and agencies, national disaster management agencies, emergency services (eg, fire department, police, ambulance), and military service [48]. The third stakeholder is health institutions, including the Ministry of Health and health authorities, health-related nongovernment organizations (NGOs), health care providers, hospitals and other health care facilities, the health workforce, and private sector health organizations and professionals [48]. Other groups include universities and research institutes, nongovernment and volunteer groups, media, social media, new media, community influencers, and the private sector [48]. Finally, regional and international organizations include WHO and international NGOs [48].

This research focuses on community stakeholders affected by health hazards. Individuals can contribute to community-level surveillance, household preparedness, first-aid training, and emergency response [48]. Their active engagement in all aspects of EDM activities is vital [48]. To encourage active participation from the community, EDM needs to implement effective risk communication to attract attention and encourage user compliance. A warning is a risk communication used to inform people about a hazard so that accidents, injuries, or unwanted consequences can be avoided [49,50]. The purpose of warning messages is to draw the attention of people at risk, to reduce the time it takes to understand the warning, and to guide people to take appropriate protective measures [51,52,53]. In this case, risk communication serves as a component of disaster control and strategies to reduce the health, economic, and psychosocial impacts of major disaster events [40]. Risk communication is vital in all EDM phases, especially the response phase. The next section maps EDM phases and activities to EDMS features.

#### Emergency and Disaster Management System

Various studies have used different terms to describe the EDMS features for each of the 4 EDM phases. The term “emergency management system” is used by the United Nations [4] and Landau [54], which refers to a system that works for emergency planning, control, and reduction. The term “disaster management system” is used by Hannan et al [55] and Currion et al [56] as a system that aims to manage relief operations, recovery, and rehabilitation. The term “emergency response system“ is used by Amailef and Lu [57] and refers to a system that supports communications, data gathering, data analysis, and decision-making during the response phase. Meanwhile, research by Edwards et al [58] and Malizia et al [59] uses the term “emergency notification system,” which refers to a system that aims to broadcast information to as many devices as possible. Other studies by Ouyang et al [60] and Bonaretti and Fischer-Preßler [61] use the term “early warning system,” which aims to disseminate warnings to a threatened population before or at an early stage of hazardous events. In the context of biological hazards that threaten public health, the terms “digital contact tracing” [14,62] and “contact-tracing application” [34,63] are used to refer to a system that monitors and tracks infection and provides immediate support and information during pandemics.

Tables 1-4 map the activities and system features for each EDM phase and the stakeholders involved according to previous studies. The stakeholders consist of the community, governments, health institutions, other groups, and regional and international organizations. Tables 1-4 also show the stakeholders' roles in each feature with the responsible, accountable, consulted, and informed (RACI) matrix. In this study, the RACI matrix was used to analyze stakeholders and their level of involvement and authorization in each feature of the EDMS. Because this research focused on community users, the RACI matrix showed which features require involvement from community users. From the 4 stages of EDM, 19 main activities and 42 EDMS features were identified. Among all the features identified, there were 27 features that require involvement from the community users that the other stakeholder groups should pay attention to in order to encourage user compliance with the warning messages.

Based on Tables 1-4, community users can act as actors who are informed by or responsible for several features in an EDMS. Before a hazard occurs, community users can receive information about policies and strategies, potential hazards, and various types of education to increase their awareness of emergencies and disasters. When a hazard occurs, community users need information about the location of shelters, evacuation routes, and regular reports on ongoing disasters. During the response and recovery phases, community users play a more important role because in addition to obtaining information, they are also responsible for being actively involved in disaster management efforts. They must actively report disaster victims and missing persons, report tracing data and lab results, report damage to infrastructure, and request medical assistance and treatment. Community users who actively use EDMS features are encouraged to comply with the protocols and measures to deal with emergencies and disasters. Therefore, the design of these features is essential to drive user compliance.

**Table 1:** System features and key stakeholders of an EDMS^a^ for the mitigation (MT) activity.

Activity code and system feature		Key stakeholders				
		Co^b^	G^c^	H^d^	O^e^	Ro^f^
**MT1**						
	MT1.1. Manage document repositories [64]	N/A^g^	A^h^	C^i^	N/A	N/A
	MT1.2. Create maps [64]	N/A	A	C	N/A	N/A
	MT1.3. Disseminate information to increase public awareness [56]	I^j^	A	I	I	N/A
**MT2**						
	MT2.1. Identify possible hazards [20]	I	A	C	I	N/A
	MT2.2. Assess vulnerability and impact of the hazard [20,64]	I	A	C	I	N/A
	MT2.3. Assess local capacity capability [20]	N/A	A	C	I	N/A
**MT3**						
	MT3.1. Develop mitigation strategy and policy [20,64]	I	A	C	I	N/A
	MT3.2. Manage data on zonation, land use, and hazard-resistant infrastructure [18]	I	A	N/A	I	N/A
**MT4**						
	MT4.1. Monitor and report the potential hazard [19,43]	I	A	R^k^	I	N/A
	MT4.2. Manage vaccination system data [19]	I	A	R	I	N/A

^a^EDMS: emergency and disaster management system.

^b^Co: community.

^c^G: governments.

^d^H: health institutions.

^e^O: other groups.

^f^Ro: regional and international organizations.

^g^N/A: not applicable.

^h^A: accountable.

^i^C: consulted.

^j^I: informed.

^k^R: responsible.

**Table 2:** System features and key stakeholders of an EDMS^a^ for the preparedness (PR) activity.

Activity code and system feature		Key stakeholders				
		Co^b^	G^c^	H^d^	O^e^	Ro^f^
**PR1**						
	PR1.1. Manage a hazard database [64]	N/A^g^	A^h^	I^i^	I	I
	PR1.2. Manage available resources and personnel database [64]	N/A	A	I	I	I
**PR2**						
	PR2.1. Create an emergency and disaster plan [18]	N/A	A	C^j^	N/A	C
	PR2.2. Manage multiorganizational partnership and communication [64]	N/A	A	R^k^	R	R
	PR2.3. Manage financial resources [18,19,43]	N/A	A	N/A	N/A	N/A
**PR3**						
	PR3.1. Manage personnel recruitment and allocation [18]	N/A	A	I	I	I
	PR3.2. Manage training and scenario data [18,19,43,64]	N/A	A	I	I	I
**PR4**						
	PR4.1. Manage supplies and equipment procurement and storage [18,19,43,56]	N/A	A	N/A	N/A	N/A
**PR5**						
	PR5.1. Monitor, detect, and report the potential hazard [19,43]	N/A	A	C	I	C
	PR5.2. Analyze spatial data [64,65]	N/A	A	C	N/A	C
	PR5.3. Disseminate an early warning [64,66]	I	A	A	I	A

^a^EDMS: emergency and disaster management system.

^b^Co: community.

^c^G: governments.

^d^H: health institutions.

^e^O: other groups.

^f^Ro: regional and international organizations.

^g^N/A: not applicable.

^h^A: accountable.

^i^I: informed.

^j^C: consulted.

^k^R: responsible.

**Table 3:** System features and key stakeholders of an EDMS^a^ for the response (RS) activity.

Activity code and system feature		Key stakeholders				
		Co^b^	G^c^	H^d^	O^e^	Ro^f^
**RS1**						
	RS1.1. Manage shelter and response organization data [56,64]	I^g^	A^h^	R^i^	R	I
	RS1.2. Manage missing person and victim data [56,64]	R	A	R	R	I
	RS1.3. Track personnel location [64]	I	A	R	R	I
**RS2**						
	RS2.1. Monitor and report the ongoing hazard [19,43]	I	A	R	I	C^j^
	RS2.2. Disseminate the warning and notification [18,19,43]	I	A	I	I	I
	RS2.3. Share timely, credible, and actionable information to the public through various channels (eg, mass media, social media) [19]	I	A	I	I	I
**RS3**						
	RS3.1. Manage evacuation data [67]	I	A	R	R	I
	RS3.2. Manage request data to deliver assistance and aid supplies for threatened populations and field respondents [19,56,64]	R	A	R	R	I
**RS4**						
	RS4.1. Manage tracing data [21]	R	A	R	I	C
	RS4.2. Manage first-aid and medical treatment data [20]	R	A	R	I	C
	RS4.3. Manage laboratory test data [20]	R	A	R	I	C
**RS5**						
	RS5.1. Manage situational reports related to infrastructure damage [68,69]	R	A	N/A^k^	R	I

^a^EDMS: emergency and disaster management system.

^b^Co: community.

^c^G: governments.

^d^H: health institutions.

^e^O: other groups.

^f^Ro: regional and international organizations.

^g^I: informed.

^h^A: accountable.

^i^R: responsible.

^j^C: consulted.

^k^N/A: not applicable.

**Table 4:** System features and key stakeholders of an EDMS^a^ for the recovery (RC) activity.

Activity code and system feature		Key stakeholders				
		Co^b^	G^c^	H^d^	O^e^	Ro^f^
**RC1**						
	RC1.1. Collect data on the hazardous event and impacted population [64]	N/A^g^	A^h^	C^i^	C	C
	RC1.2. Create reports related to the responses [64]	I^j^	A	I	I	I
**RC2**						
	RC2.1. Assess infrastructure and lifeline service damage [69]	N/A	A	N/A	I	I
	RC2.2. Manage data of infrastructure and lifeline services rebuilding [18]	I	A	N/A	I	C
**RC3**						
	RC3.1. Manage continuous tracing or mapping of case data [21]	R^k^	A	R	I	C
	RC3.2. Manage continuous medical treatment and mental health care [20,64]	R	A	R	I	C
	RC3.3. Manage continuous laboratory testing data [20]	R	A	R	I	C
**RC4**						
	RC4.1. Manage continuous monitoring and reporting of hazards [19,43]	I	A	R	I	C
**RC5**						
	RC5.1. Manage lessons learned data [64]	I	A	C	I	C

^a^EDMS: emergency and disaster management system.

^b^Co: community.

^c^G: governments.

^d^H: health institutions.

^e^O: other groups.

^f^Ro: regional and international organizations.

^g^N/A: not applicable.

^h^A: accountable.

^i^C: consulted.

^j^I: informed.

^k^R: responsible.

#### Health Emergency and Disaster Management System

All the features analyzed in the previous section are used in a general EDMS dealing with all types of emergencies and disasters. To find out how these features have been used to address health-related disasters, we investigated their application to Health EDMS, an EDMS that is specifically used to respond to and cope with hazardous events that threaten public health. During the COVID-19 pandemic, Health EDMS was used by multiple countries to assist in contact tracing. Contact tracing is a control measure to prevent further disease transmission [70]. When someone tests positive for COVID-19, that person must be quarantined or self-isolated. After that, the contact-tracing process is carried out by identifying the person's close contacts and advising them to take precautionary self-isolation [71]. Close contact means direct face-to-face contact with an infected person or confirmed case [71]. Various countries have developed contact-tracing mechanisms with technology support. A CTA allows devices to communicate through Bluetooth technology, GPS, wireless technology, and sensors [71]. A CTA has features to automate contact-tracing activities and other features to disseminate information and provide medical care [70].

In this study, the discussion of Health EDMS will focus on a CTA because a CTA is a clear example of a technology application that deals with health emergencies and disasters. As a self-administered warning system, CTAs have been widely used in more than 50 countries [72]. A CTA is implemented when COVID-19 emerges in order to respond to and recover from the pandemic [16]. Therefore, the system features are geared more toward the response and recovery phases than the mitigation and preparedness phases. Table 5 displays a list of features for response and recovery phases in some CTAs implemented by different countries. The countries shown were selected based on the number of COVID-19 cases: high (the United States and India), medium (the United Kingdom and France), and low (Singapore and Switzerland) [73]. In addition to the number of cases, these countries were also chosen to represent the Americas, Europe, and Asia regions. The CTAs selected are available on Apple App Store and Google Play Store with the most significant number of users, free of cost, and launched and supported by the governments of selected countries [73]. The CTA features in the selected countries were based on the information provided by Alanzi [16] and Blasimme et al [17].

There are 6 CTAs reviewed in Table 5. PathCheck SafePlace is a CTA developed in the United States that aims to integrate people and health departments to prevent the spread of COVID-19 by sharing information [16]. The application was developed by the Massachusetts Institute of Technology, TripleBlind, and a nonprofit organization called PathCheck Foundation [16]. Aarogya Setu is an official CTA launched by the Indian government with more than 1.4 million users [16]. NHS COVID19 is a CTA launched by the National Health Service (NHS) Test and Trace in the United Kingdom [16]. Although this application does not store personal information, it still collects location information for track and trace purposes [16]. TousAntiCovid is implemented in France using a centralized IT architecture where data are stored on centralized servers run by the national health authorities [17]. Users must enter their personal information to report a positive test, which triggers a notification to other users [17]. The health authorities in France send an exposure code to users via email and post [17]. In contrast to TousAntiCovid, SwissCovid implements a decentralized protocol like in other European countries, such as the United Kingdom [66]. Users must contact health authorities to activate an exposure code after receiving a notification of a positive test result [66]. Another application, TraceTogether, is an official CTA launched by the Singapore government [16]. This application emphasizes user privacy by using anonymous IDs and giving users the right to delete their data [16].

**Table 5:** CTA^a^ features.

EDMS^b^ feature code			PathCheck SafePlace (United States)		Aarogya Setu (India)		NHS^c^ COVID19 (United Kingdom)		TousAntiCovid (France)		Trace Together (Singapore)		SwissCovid (Switzerland)
**Response (RS)**													
	RS1.1	Unknown		Unknown		Unknown		Unknown		Unknown		Unknown	
	RS1.2	Unknown		Unknown		Unknown		Unknown		Unknown		Unknown	
	RS1.3	Unknown		Unknown		Unknown		Unknown		Unknown		Unknown	
	RS2.1	Yes		Yes		Yes		Yes		Yes		No	
	RS2.2	Yes		Yes		Yes		Unknown		Yes		Unknown	
	RS2.3	Yes		Yes		Yes		Yes		Yes		No	
	RS3.1	Unknown		Unknown		Unknown		Unknown		Unknown		Unknown	
	RS3.2	Yes		Yes		No		Unknown		No		Unknown	
	RS4.1	Yes		Yes		Yes		Yes		Yes		Yes	
	RS4.2	Yes		Yes		Yes		Unknown		No		Unknown	
	RS4.3	Yes		Yes		Yes		No		Yes		No	
	RS5.1	Unknown		Unknown		Unknown		Unknown		Unknown		Unknown	
**Recovery (RC)**													
	RC1.1	Unknown		Unknown		Unknown		Unknown		Unknown		Unknown	
	RC1.2	Unknown		Unknown		Unknown		Unknown		Unknown		Unknown	
	RC2.1	Unknown		Unknown		Unknown		Unknown		Unknown		Unknown	
	RC2.2	Unknown		Unknown		Unknown		Unknown		Unknown		Unknown	
	RC3.1	Yes		Yes		Yes		Yes		Yes		Yes	
	RC3.2	Yes		Yes		Yes		Unknown		No		Unknown	
	RC3.3	Yes		Yes		Yes		No		Yes		No	
	RC4.1	Yes		Yes		Yes		Yes		Yes		No	
	RC5.1	Unknown		Unknown		Unknown		Unknown		Unknown		Unknown	
Total “yes,” n			11		11		10		5		8		2

^a^CTA: contact-tracing application.

^b^EDMS: emergency and disaster management system.

^c^NHS: National Health Service.

Based on Table 5, at the most, the CTAs implemented 11 EDMS features in 5 EDM activities: response (RS)2, RS3, RS4, recovery (RC)3, and RC4. Most CTAs implemented all 3 features in RS2 to monitor and report the ongoing hazard; disseminate notifications and warnings; and provide timely, credible, and actionable information to the public. The CTAs also provide data visualization that enables users to monitor the ongoing status of the COVID-19 cases in their areas. Some CTAs (ie, PathCheck SafePlace, and Aarogya Setu, provide query resolution feature as part of RS3, allowing users to request help and ask questions through the applications [16].

Medical care provision (RS4) is 1 of the priority features in CTAs. CTAs provide automatic contact-tracing tools integrated with location mapping through GPS. Aarogya Setu integrates these tools with an electronic pass (e-pass) in transportation and public places [16]. CTAs also provide users with self-assessment tools to check their health status and exposure to COVID-19. Health institutions provide laboratory check reports to users through their handheld devices. Some CTAs(ie, PathCheck SafePlace and Aarogya Setu) also come with online consultation and appointment scheduling with health facilities [16]. The medical care and surveillance activities continue to the recovery phases in RC3 and RC4.

In the previous section, there were 27 EDMS features that involved the user community. Meanwhile, Table 5 shows that only 11 have been implemented in Health EDMS, mainly functioning to monitor and report ongoing hazards and provide medical care and logistical support. Health EDMS does not implement all the features at the mitigation and preparedness stages. Several features in the response and recovery phases are also not implemented because they are not in accordance with the scope of health-related hazards, such as shelter data management, reporting of victims and missing persons, and recording of infrastructure damage. In Health EDMS, all the measures need to be complied with by the users to make them effective in controlling the pandemic [38,40]. When surveillance and monitoring tools detect hazardous conditions, Health EDMS disseminates notifications and warnings to users. User compliance with notifications and warnings determines the success of Health EDMS. The following section discusses user compliance in the information system (IS) context.

#### Compliance

Compliance refers to the agreement with the expectations stated in the rules, standards, proposals, requests, orders, or suggestions [74]. It is also defined as a relationship consisting of the power used by superiors to control subordinates and subordinates' orientation to this power [75]. Compliance can include approval as well as obedience [74]. In health care, compliance is conceptualized as a cognitive-motivational process of personal attitudes and intentions, behaviors, and the outcome of patient-practitioner interactions [76]. It also involves professional power over the patient [76]. Compliance is not based solely on an individual risk-benefit assessment but also involves relationships between commanders and followers and between organizations and members [38].

Research on compliance has been investigated in various fields, and theories regarding compliance also come from multiple domains, such as psychology, criminology, health, management, and organization [77]. In the organizational context, business dynamism and IT advancements encourage companies to frequently update their IS and its usage policy [78]. In this case, users need to maintain IS compliance behavior due to the interdependent nature of tasks that need to be accomplished through IS [78]. Social (trust and support) and performance (discipline and stretch) management influences collective compliance with IS [78]. Compliance also has been widely studied in IS security policy research [38]. Employees are willing to comply with IS security policy if they feel they have the capacity to carry out a security task, have a positive attitude in carrying it out, and see others performing the same security task [77]. When they encounter a security threat, they evaluate the threat and coping behavior to decide whether to comply [77]. Employee compliance with IS and its usage policy is critical to achieving IS objectives in supporting business operations [78].

Health EDMS differs from other ISs as it is used in life-critical and time-sensitive situations, often with limited resources. Immediate compliance with the health notifications and warnings is essential to save lives [38]. When a person receives a warning, the warning response can be sequenced as follows: listen to the warning, understand the content of the warning message, believe that the warning is credible and accurate, personalize the warning, confirm the warning, and respond by taking protective action [79]. Previous studies have shown that responses to warning messages are influenced by the type of hazard, the message and its source, the medium or channel through which the message is communicated, the characteristics of the recipient, and situational or environmental factors [51,79]. We conducted a systematic literature review to map the extant studies on user compliance with Health EDMS.

## Methods

### Search Strategy

A systematic literature review is a secondary study conducted to identify, evaluate, and interpret all available research relevant to a particular research question, topic area, or phenomenon of interest [80]. A systematic literature review summarizes the existing research insights, identifies research gaps, suggests areas for further investigation, or provides a framework to position new research activities [81]. Our systematic review was conducted using PRISMA 2020 guidelines [42]. The PRISMA checklist can be seen in Multimedia Appendix 2.

The search was conducted using the online databases Scopus, ScienceDirect, ProQuest, IEEE, and PubMed. The keywords or search strings used to search the papers were (“emergency” OR “disaster” OR “response” OR “notification” OR “warning” OR “alert” OR “tracing”) AND (“system” OR “apps” OR “application”) AND (“compliance”). The search was conducted for journal papers published between January 2000 and February 2022.

### Inclusion and Exclusion Criteria

The inclusion criteria were the review guidelines for study selection, as displayed in Table 6: be published in a journal, have full text available, and be written in English. In addition, in accordance with the objectives of this study, the papers had to discuss compliance with the warning or alert from Health EDMS and focus on individual perceptions or behaviors on Health EDMS. Therefore, papers with related terms, such as “emergency management system,” “disaster management system,” “emergency notification system,” “emergency warning system,” “emergency response system,” and “contact-tracing application,” were also included in this review. We did not include the keywords “mobile health” or “mHealth” because these applications are not used specifically for health emergencies and disasters but are for everyday use, which was not the scope of this research. This study was limited to a review of previous research on compliance from a user perspective and did not include the technical design of Health EDMS.

**Table 6:** Inclusion and exclusion criteria.

	Inclusion criteria	Exclusion criteria
Paper type	Journal paper	Other than journal paper (eg, conference paper, book, editorial)
Language	English	Other than English
Publication date	January 2000-February 2022	Before January 2000 and after February 2022
Topic	User compliance with Health EDMS^a^	Compliance with health behavior without the use of the system or application, organization compliance, and technical design of Health EDMS

^a^EDMS: emergency and disaster management system.

### Study Selection

The study selection was carried out as follows:

- Step 1: The keyword or search string was searched in the aforementioned online databases. We limited the search to the abstract, title, or keyword fields. Duplicate records were removed.
- Step 2: The title and abstract of the identified papers were reviewed based on the inclusion criteria. Papers that did not meet inclusion criteria were removed.
- Step 3: The remaining papers were read in full to determine whether they met the inclusion criteria.

### Data Items and Synthesis

The data extraction process aimed to identify relevant information from the included studies that pertained to our research question. This process included producing a Microsoft Excel data sheet consisting of key aspects related to the research aim. The following data were extracted from each publication: title, author(s), year of publication, name of the journal, country, topic, research question or objective, factor(s), method, recommendation, finding, and research gap. Each paper's full text was read, and the research data were entered into the Excel sheet. Once the extraction was completed, the Excel sheet was reviewed, and then the findings of the studies were analyzed to answer the research question. The results of the review are discussed in the next section.

## Results

### Review Process

**Figure 2:**
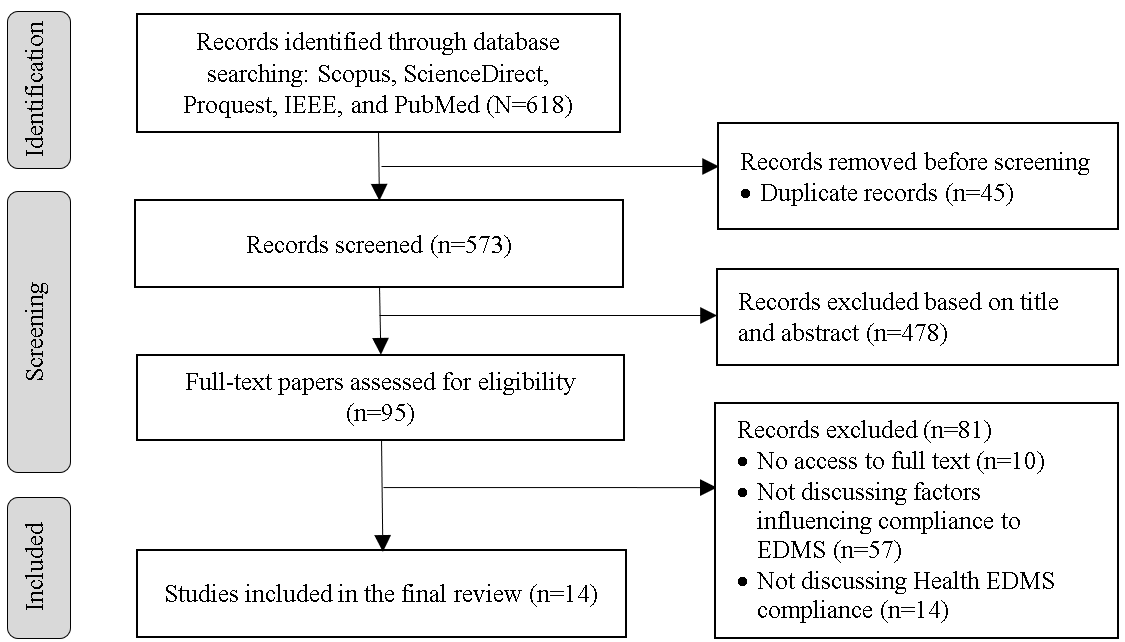
PRISMA flow diagram. EDMS: emergency and disaster management system; PRISMA: Preferred Reporting Items for Systematic Reviews and Meta-Analyses.

The selected electronic databases were searched following the previously explained search strategy. In total, 618 papers were retrieved. Next, duplicate papers were removed, resulting in 573 (92.7%) papers. The papers' titles and abstracts were reviewed by applying the inclusion criteria. After removing the papers that did not fulfill the inclusion criteria, we were left with 95 (16.6%) papers. Next, the papers' full texts were read to ensure they covered the predefined scope. This step resulted in 14 (14.7%) selected papers. A summary table of the characteristics of the included studies is provided in Multimedia Appendix 3. Figure 2 shows the review process using PRISMA guidelines.

### Study Characteristics

**Figure 3:**
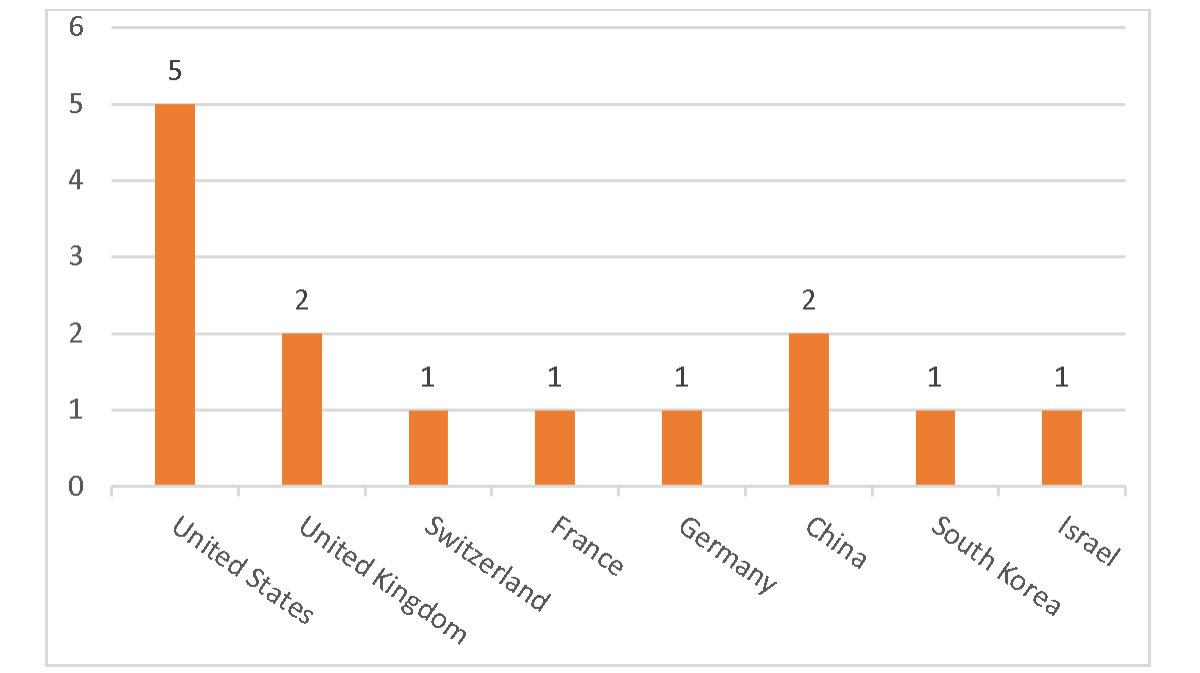
Distribution of papers based on country.

The included papers showed that Health EDMS research was conducted in the United States (n=5, 35.7%), the United Kingdom (n=2, 14.3%), China (n=2, 14.3%), Switzerland (n=1, 7.1%), France (n=1, 7.1%), Germany (n=1, 7.1%), South Korea (n=1, 7.1%), and Israel (n=1, 7.1%). Figure 3 shows the distribution of the papers by country.

From 2004 to 2022, 14 studies on user compliance with Health EDMS were found. There were relatively few studies before 2021. Research on Health EDMS compliance increased rapidly in 2021 (there were 7, 50.0%, studies) due to the COVID-19 pandemic. The COVID-19 pandemic has led to many studies on user compliance with CTAs, EDMSs for handling health-related emergencies and disasters.

Related to the sources of publications, 2 (14.3%) papers were published in the *Journal of Medical Internet Research* and 1 (7.1%) paper each was published in the following journals: *MIS Quarterly*, *AIS Transactions on Replication Research*, *Applied Ergonomics*, *BMJ Open*, *Disaster Medicine and Public Health Preparedness*, *Disasters*, *Frontiers in Psychology*, *Human Factors*, *Humanities and Social Sciences Communications*, *International Journal of Disaster Risk Reduction*, *Public Health*, and *Public Relations Review*. These papers used the quantitative research method (n=11, 78.6%), mixed method (n=1, 7.1%), and conceptual method (n=2, 14.3%). The papers that applied quantitative methods used surveys (n=7, 50.0%) and experiments (n=4, 28.6%) to collect data. The 1 (7.1%) paper that applied a mixed method used a combination of experiments (quantitative) and focus group discussions (qualitative). In addition, 2 (14.3%) conceptual papers developed arguments about concept associations.

### Theoretical Lens of User Compliance with Health EDMS

The theories used in Health EDMS compliance research (Table 7) include the protective action decision model (PADM), Etzioni's compliance theory, the Health Belief Model (HBM), protection motivation theory (PMT), social amplification of risk framework (SARF), extension of the technology acceptance model (TAM2), and the theory of planned behavior (TPB). Of the 14 papers in this review, 5 (35.7%) did not use any theory to analyze compliance factors, 2 (14.3) were conceptual papers [82,83], and the others applied selected concepts or themes from previous studies. To investigate compliance with Health EDMS, Freberg [84] examined the message source and message reliability; Albrecht et al [85] examined risk perception, risk preference, and social preference; and Panchal et al [86] examined security and privacy concerns, information content, usability, and knowledge of the application. Next, we elaborate on each of the theories used in Health EDMS compliance research.

**Table 7:** Theoretical lenses to investigate compliance with Health EDMS^a^.

Theory	Papers (N=14), n (%)	Reference(s)
PADM^b^	3 (21.4)	[15,52,87]
Etzioni’s compliance theory	2 (14.3)	[38,41]
HBM^c^	2 (14.3)	[87,88]
PMT^d^	2 (14.3)	[89,90]
SARF^e^	1 (7.1)	[15]
TAM2^f^	1 (7.1)	[27]
TPB^g^	1 (7.1)	[90]

^a^EDMS: emergency and disaster management system.

^b^PADM: protective action decision model.

^c^HBM: Health Belief Model.

^d^PMT: protection motivation theory.

^e^SARF: social amplification of risk framework.

^f^TAM2: extension of the technology acceptance model.

^g^TPB: theory of planned behavior.

#### Protective Action Decision Model

The PADM is a framework for managing the societal response to environmental hazards [51]. The PADM has been applied to 3 different areas: risk communication, evacuation modeling, and long-term hazard adjustment [51]. In the PADM, decision-making begins with environmental cues, social cues, and warnings [51]. Environmental cues are signs of threat obtained from the environment, such as sight, smell, or sound [51]. Social cues are signs obtained after observing the behavior of others [51]. A warning is a message sent from an information source through a channel to the recipient [51]. The 3 triggers encourage a pre–decision-making process involving exposure, attention, and comprehension [51]. Exposure measures whether people receive information, attention measures whether people care about the information, and comprehension measures whether people understand the information [51].

Furthermore, the pre–decision-making process generates perceptions of the environmental threats, alternative protective actions, and relevant stakeholders (government, other groups, and community) [51]. These perceptions provide the basis for protective action decision-making [51]. The dependent variable of the PADM is a behavioral response that is generally in the form of information seeking, protective responses, or emotion-focused coping [51]. The actual implementation of a response depends not only on people's intentions to act but also on the physical and social conditions that can hinder or facilitate the action [51]. The final stage in the PADM is the feedback loop [51]. People attempt to confirm or contradict every warning they receive, usually by searching for additional information from different sources and channels [51].

Stakeholder perceptions and protective action perceptions have been shown to influence compliance with warnings during the avian influenza A (H7N9) outbreak [87] and the COVID-19 pandemic [15]. Meanwhile, threat perception has not been shown to affect warning compliance based on COVID-19 research in Germany and China [15,52]. The use of protective measures can reduce threat appraisal, making people feel less likely to be infected. However, the duration of the pandemic, the extended and repeated warnings, and the familiar feeling of the disease resulted in information fatigue that undermined the perceived threat [52]. Among all the theories in Table 7, the PADM is the only theory that focuses on the human response to threats [51]. The PADM also specifies prompts for action, which are important for encouraging engagement in health-related behaviors [87]. However, for slow-moving and long-term health-related hazards, the PADM has not been able to predict changes in threat perceptions.

#### Etzioni's Compliance Theory

Etzioni's compliance theory states that there are 2 parties to a compliance relationship: an actor who has power and another actor who responds to the power (subordinated actor) [91]. Power can be differentiated into 3 types according to the means used to make the subject comply [91]. Coercive power applies physical sanctions, force, and fear to control lower-level participants [91]. Remuneration power applies material resources and rewards to control others [91]. However, normative power is the provision and manipulation of symbolic rewards and deprivations [91]. The effectiveness of each power in obtaining the cooperation of subordinates depends on their involvement [91].

The subordinated actor can have 3 kinds of involvement: alienative, calculative, and moral [91]. Alienative involvement leads to intense negative orientation, while calculative involvement can lead to positive or negative orientation [91]. However, moral involvement, also known as commitment, leads to intensely positive orientation [91]. Moral involvement can be based on pure commitment (based on the internalization of norms) and social commitment (based on pressure from primary groups and their members) [91]. Subsequently, combining 3 types of power and 3 types of involvement produces 9 kinds of compliance [91]. However, 3 kinds of compliance are more frequently found in practice [91]. These are the relationships between coercive power and alienative involvement, remuneration power and calculative involvement, and normative power and moral involvement [91]. These compliance types are called congruent types [91]. This happens when the subordinate's involvement type is the same as the types that want to be generated by organization power [91]. The congruent types are more frequent because they are more effective to implement.

Etzioni's compliance theory has been adapted to campus emergencies [38] and health care–related emergencies [41]. Both studies were designed to accommodate emergency notifications sent by normative organizations, such as the campus and the government [38,41]. Normative power was adapted as the subjective norm, while coercive power and remuneration power were adapted as a perceived security threat and a perceived financial threat, respectively [38,41]. The results of the 2 studies are similar and show that subjective norms are the most important factor promoting immediate compliance in health-related hazard scenarios [38,41].

#### Health Belief Model

The HBM is a conceptual model for understanding why individuals do or do not perform various actions related to health behavior [92]. The HBM defines 6 different constructs. These constructs focus on individual representations of threat perceptions and evaluation of health behaviors [93]. Threat perception is defined as 2 central beliefs: perceived susceptibility to health problems and perceived severity of disease consequences [93]. Health behavioral evaluation also consists of 2 constructs: perceptions of the benefits or efficacy of the recommended health behaviors and perceptions related to the barriers to enacting these behaviors [93]. In addition, the HBM proposes that cues to action can activate health behaviors when individuals hold appropriate beliefs [92]. Cues to action can be individual perceptions of symptoms, social influences, and health education campaigns [93]. In a later version of the theory, individual health motivations are also included as constructs that drive health behavior actions [93].

The HBM has been used to analyze individual compliance with warnings to take recommended protective actions during the H7N9 [87] and COVID-19 [88] outbreaks. The perceived risk or perceived vulnerability positively influenced compliance intentions in the H7N9 outbreak [87]. Meanwhile, all HBM constructs except health motivation were used by Guillon and Kergall [88] and were shown to affect compliance to protective measures and CTAs during the COVID-19 pandemic. However, the HBM does not consider the effect of information on protective behavior, even though risk information has been seen as an essential driver of risk perception and behavioral response [87].

#### Protection Motivation Theory

In a hazardous event, the increased fear of the individual also increases their intention to take action [90]. The PMT describes the social and cognitive processes that underlie protective behavior [94]. The PMT proposes that people protect themselves based on threat appraisal and coping appraisal [94]. Threat appraisal refers to the perceived likelihood that the event will occur and have negative consequences [94]. Threat appraisal is a person's assessment of the estimated severity of the disease (perceived severity) and estimates the likelihood of contracting the disease (perceived susceptibility) [95]. In addition, the PMT also states that fear arousal indirectly affects attitudes and behavior changes through the perceived severity of danger [95]. However, coping appraisal consists of the individual's expectation that implementing the recommendations will eliminate the threat (response efficacy) and the belief that they can successfully carry out the recommended actions (self-efficacy) [95]. Thus, if individuals conclude that a threat will affect them, they will be more motivated to protect themselves and initiate or continue certain self-protective behaviors [94].

In Health EDMS compliance studies, the PMT has been used in campus emergency notification systems [90] and emergency alerts during the COVID-19 pandemic in South Korea [89]. However, the PMT constructs cannot explain rapid compliance with health advisory warnings on campus. Instead, the PMT can better explain compliance in fast-evolving scenarios, such as robberies, active shooters, and building fires. In the COVID-19 alert study, the PMT construct was used as a mediating factor between reading text messages and practicing preventive behaviors. Response efficacy was the only variable driving compliance to warnings, whereas the perceived risk was insignificant. Based on these 2 studies [89,90], the PMT constructs are less predictive of explaining compliance in slower-developing scenarios, such as health-related hazards.

#### Social Amplification of Risk Framework

SARF describes a dynamic process for understanding how risk is perceived when communicated to the community [15]. SARF conceptualizes that individual and social perceptions of risk and risk behavior can be shaped, enhanced, or attenuated when there is an interaction between hazardous events and psychological, social, institutional, and cultural processes [96]. In this case, the IS and the characteristics of the public response can form a social amplification that determines the nature and magnitude of the risk. ISs can amplify risk events in 2 ways: by intensifying or attenuating the signals that individuals and social groups receive about risk and by filtering out the many signals related to risk attributes and their importance [96].

Amplification occurs in 2 stages: when transferring information about the risk and when the community responds to the information [96]. Signals about risk are processed by individual and social amplification stations, including scientists communicating risk assessments, news media, cultural groups, and interpersonal networks [96]. Reinforced risk leads to a behavioral response, resulting in secondary impacts, such as financial loss, organizational change, and physical risk [96]. Communication is at the core of SARF, as individuals are most often exposed to risky information through the media or discussions with others [15]. In this manner, risk amplification allows different “amplification stations” to compete for public attention and influence how the public perceives and responds to risk [15].

SARF has been used to investigate compliance behavior in China during the COVID-19 pandemic [15]. One variable from SARF, namely information interaction, is used to examine how interactions between individuals can shape risk perceptions [15]. Information interaction significantly influences risk perception and preparedness intentions, while also serving as a mediator for warning [15]. Of the 2 stages of amplification in SARF, this study only analyzed amplification when the public responds to information [15]. Other SARF variables have not been found in Health EDMS compliance studies.

#### Extension of the Technology Acceptance Model

TAM2 extends the original TAM by including additional key determinants of perceived usefulness and usage intention constructs: social influence and cognitive instrumental process [97]. The social influence processes consist of subjective norms, voluntariness, and image [97]. Subjective norms are added as determinants considering that people can choose to perform a behavior if they believe their important references think they should [97]. TAM2 distinguishes between mandatory and voluntary use arrangements by adding voluntariness as a moderating variable [97]. In addition, TAM2 theorizes that social influences can affect how users judge the system's image (ie, whether the system can increase their status in a social system) [97]. The elevated status leads to increased power and influence that eventually provides a general basis for greater productivity (perceived usefulness) [97]. TAM2 also investigates how these determinants' effects change with increasing user experience over time with the target system [97]. As cognitive instrumental processes, TAM2 includes job relevance, output quality, and result demonstrability constructs. TAM2 shows that the influence of cognitive instrumental processes is not influenced by experience over time [97].

Research on CTAs in the United Kingdom found that most TAM2 constructs significantly affect user behavior to download the application and comply with notifications [27]. The trust factor was added and proved to be significant [27]. Compliance with the notification was fairly high, but there were issues surrounding trust and understanding of the application's features that hindered the adoption of the CTA [27]. Moreover, Dowthwaite et al [27] stated that users would likely delete the CTA when they are frustrated by a notification from the application (output quality) and do not understand how their data would be used (result demonstrability). Although TAM2 has described the factors that influence compliance with Health EDMS, it is still unclear what level of experience, output quality, and result demonstrability can positively affect compliance.

#### Theory of Planned Behavior

The TPB was first introduced as a development of the theory of reasoned action (TRA) and has been most widely adopted in the research on motivations for human behavior. The TPB explains that behavior is influenced by intention and intention is determined by 3 types of beliefs: attitudes, subjective norms, and perceived behavioral control [98]. Attitude is a comprehensive evaluation of the implementation of behavior [98]. Subjective norms are individual perceptions of other people's expectations considered important about certain behaviors [98]. Meanwhile, perceived behavioral control is an individual's perception of how easy or difficult it is to do something [98]. It can also be interpreted as the resources and opportunities available to a person to encourage them to perform a behavior [98]. The TPB has been applied in studies of ISs, organizations, and user populations.

The TPB is 1 of the most influential theories in disaster and emergency preparedness planning. The TPB has been used to analyze the factors that affect rapid compliance with emergency notifications in 7 emergency scenarios in a campus: robbery, active shooter, building fire, hazardous material, riot/violent protest, air quality advisory, and health advisory [90]. Attitude significantly affected compliance across all scenarios, while subjective norms significantly affected almost all scenarios except the active shooter [90]. However, perceived behavioral control was only significant in the air quality advisory scenario [90].

### Factors Affecting User Compliance With Health EDMS

This section summarizes the factors affecting compliance with Health EDMS from the selected papers. People can comply immediately or verify and then comply after receiving a warning [38]. Verification is performed by contacting other people or seeking information from other media and channels [41]. In this case, various features of Health EDMS, such as warning and notification (RS2.2), interactive messaging (RS2.3), or video and media (RS2.3), can facilitate remote verification to increase compliance levels [41]. Other technological factors, such as system design, including communication channels, public education, usability, and information content, also affect warning compliance [78].

In addition, individual characteristics and social influence play a significant role in driving compliance [78,85,90]. The individual evaluates the situation to build their belief about whether compliance with an emergency message yields a valuable outcome [90]. They also consider their expectations from influential people in their lives, social pressures, and cultural norms about behavior [90]. The factors affecting compliance with Health EDMS can be categorized into 3 main groups: individual, technology, and social.

Sorensen [79] stated that warning compliance is influenced by sender and receiver factors, situational factors, and social contacts. Upon receiving the warning, individuals make decisions based on their judgment of the message source, their ability to understand the message and implement countermeasures, and the personal situation they experienced. The sender and receiver are included in the category of individual factors. Moreover, compliance results from interactions between users and other parties who provide warnings. In this study, the interaction is described in the form of social factors consisting of situational factors and social contact. Sorensen [79] also stated that system design can affect the response to warnings.

From the literature reviewed, our study identified 14 individual, 10 technological, and 4 social factors that influenced users' compliance with Health EDMS. Most of them were derived from the previously discussed behavior theories, while others were added by the researchers without referring to any particular theory. From individual factors, perceived risk and response efficacy are the most widely used predictors of compliance. From technological factors, the most frequently used factors are warning message characteristics. Moreover, subjective norms and stakeholder perception are the most researched social factors. A summary table of the individual, technological, and social factors influencing users' compliance with Health EDMS is provided in Multimedia Appendix 4.

## Discussion

### Principal Findings

EDM is 1 of the most challenging management tasks because decisions must be made in a short time, under a rapidly changing environment, with unique situations for each incident, and involve high operational costs [64]. In this case, an IS (EDMS) can be used to support decision-making. Each stage in EDM (ie, mitigation, preparedness, response, and recovery) requires a different type of EDMS feature in terms of the problem to be solved, the stakeholders involved, the need to provide real-time data, presentation of the data to users, and the technological sophistication required [64]. This research mapped EDMS features from various literatures into the main activities at each EDM stage. From the 4 stages of EDM, 19 main activities and 42 EDMS features were identified (Tables 1-4). This study also identified stakeholder roles for each feature using the RACI matrix, which will be helpful during EDMS development. The list of features presented in this study is expected to guide governments and system developers in designing a comprehensive and integrated EDMS.

This study mapped the EDMS features into CTA functionalities. Contact-tracing strategies have been implemented worldwide, with varying degrees of success [86]. In 2020, months after COVID-19 spread, CTAs were launched in more than 50 countries [72], both official and unofficial, to respond to the pandemic. This study found that at the most, a CTA implements 11 EDMS features for the response and recovery phases. For the response phase, a CTA provides features to monitor and report ongoing hazards, disseminate notifications and alerts, provide information to the public, manage request data, manage contact-tracing data, manage medical care, and manage laboratory test data. When the number of infected cases decreases and activities return to normal, a CTA can still be used in the recovery phase to support ongoing medical care and monitoring activities. An important feature that needs to be implemented in the recovery phase is the management of lessons learned data. The lessons learned can provide insight into the use of Health EDMS in the response stage [63], especially for new infectious diseases that have not been previously identified. This feature will be beneficial in determining future strategies and policies. A CTA was only implemented when the disease had spread, so there were no mitigation and preparedness functions. The system should also be equipped with these functions for further development to enable early strategic planning and build sufficient capacity for dealing with the health-related hazard.

**Figure 4:**
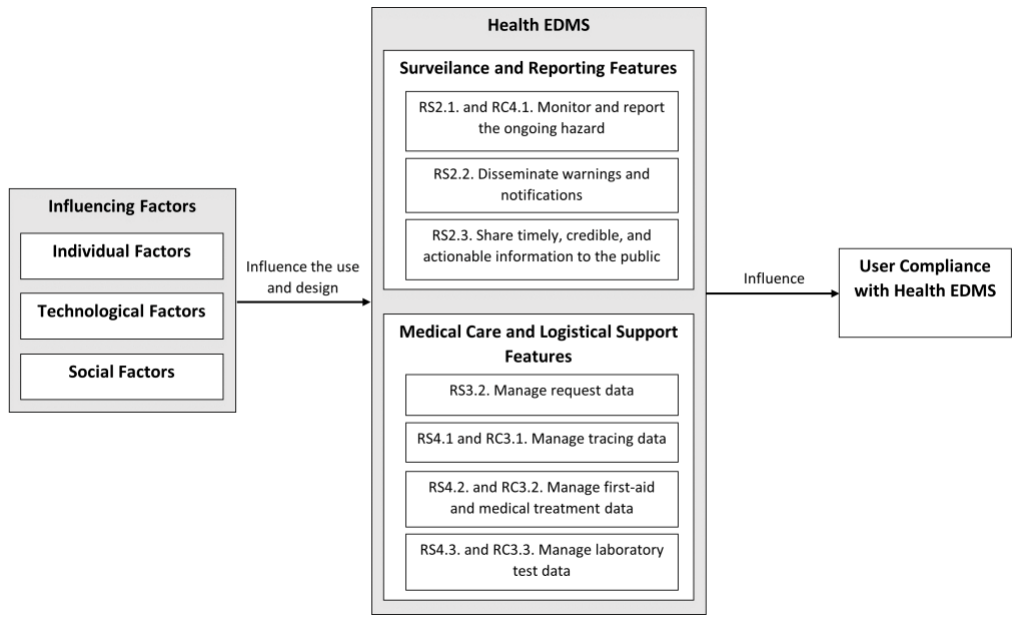
Proposed research framework. EDMS: emergency and disaster management system.

Rapid compliance with warnings is vital to save lives. The features of Health EDMS can provide information and warnings that increase the sense of urgency and become a source of verification to improve compliance levels [41]. Through a systematic literature review using the PRISMA method, this study classified factors that determine compliance with Health EDMS into individual, technological, and social factors. Based on the analysis and synthesis of the literature, we proposed a research framework showing the relationship between the influencing factors of Health EDMS feature use and design and user compliance with Health EDMS (Figure 4). The framework shows that individual, technological, and social factors influence the use and design of surveillance and reporting features, and medical care and logistical support features. Appropriate use of these Health EDMS features during health disaster events affects user compliance with Health EDMS.

In the previous explanation (Table 5), 11 features have been implemented in Health EDMS, mainly for surveillance and reporting ongoing hazards and providing medical care and logistical support. Individual, technological, and social factors influence the use of these features. Individual factors are related to perceptions of health risks, experiences, attitudes, and perceptions of Health EDMS. The perceived probability and consequences of contracting a disease can encourage someone to take protective actions in Health EDMS [85,88]. Individuals who feel the threat of an illness will seek more information about it and will always be aware of warnings and notifications from the system. Therefore, individual factors influence the use of surveillance and monitoring features of Health EDMS. During the COVID-19 pandemic, the perception of risk also encouraged the public to carry out laboratory tests and self-quarantine to ensure they did not contract the disease or infect other people [88]. They were self-encouraged to take preventive measures, such as testing, medical treatment, and requesting logistical assistance through Health EDMS.

Technological factors cover all aspects of information and technical quality. System notifications and warnings can change how individuals think and feel about threats and risks. Health EDMS must provide sufficient information to users about their possible risk exposure and the actions they should take [86]. The information quality of the system will drive the use of both feature sets in Health EDMS (ie, surveillance and monitoring, and medical care and logistical support). In addition, if users believe that Health EDMS is easy to use, relevant to their needs, and protects their privacy, they will be encouraged to use the system [27,86].

In addition to technological and individual factors, the use and design of Health EDMS are influenced by social factors. Opinions from influential people, such as family, friends, or coworkers, encourage individuals to use Health EDMS [90], for example, seeking information about hazards and viewing daily case reports. Moreover, users who believe that Health EDMS stakeholders have the expertise to manage hazards will use Health EDMS as their primary source of information and warnings [15]. Users will also ask for medical and logistical assistance through Health EDMS if they believe the stakeholders can respond quickly and effectively [15]. In designing Health EDMS, social factors can be supported by allowing users to share information to social media or messaging applications. Health EDMS must also highlight the ability of the stakeholders to overcome the health hazards [15]. Frequent use of the surveillance and monitoring features as well as the medical and logistical assistance features of Health EDMS may increase compliance. User compliance analyzed in previous research is limited to the intention to comply, not actual compliance. Future research needs to examine actual compliance to produce stronger evidence on the causal factors and effective interventions in this area.

The proposed research framework was developed based on our analysis of theories and corresponding factors affecting user compliance with Health EDMS in the existing literature. Future research can empirically examine the framework with a specific health EDMS. Collaborations with CTA providers are needed to examine actual compliance. In addition to the proposed research framework, we proposed 2 research topics on Health EDMS that require further investigation. First, Health EDMS has only implemented features to handle the response and recovery phases of EDM. The mitigation and preparedness phases of EDM are not supported by Health EDMS. Health EDMS should not only be used to respond to and cope with disasters but also be used to prevent and prepare for health-related hazards. Nevertheless, unlike natural disasters or human-induced disasters, such as forest fires, the characteristics of future health-related hazards are unknown. How to design features for mitigation and preparedness of future health-related hazards is an interesting research topic. Second, considering that a CTA is a relatively new IS implemented for a specific pandemic case, the effectiveness of CTA features in increasing user compliance with warnings has not been widely analyzed. Instead of examining a CTA as a whole, future research should investigate each feature of a CTA in depth toward increasing user compliance.

### Conclusion

Health EDMS has been implemented to cope with health-related hazards. However, the compliance rate with Health EDMS remains low [27,41]. This study reviewed previous research on user compliance with Health EDMS based on PRISMA reporting guidelines. Research on this topic increased rapidly in 2021 due to the COVID-19 pandemic. This study identified 7 theories researchers adopted when examining Health EDMS compliance behavior: PADM, Etzioni's compliance theory, HBM, PMT, SARF, TAM2, and TPB. This study also discussed the individual, technological, and social factors influencing the use of 2 Health EDMS features (ie, surveillance and reporting features, and medical care and logistical support features). During health-related hazards, community users play an important role in actively reporting disaster victims and missing persons, report tracing data and lab results, report damage to infrastructure, and request medical assistance and treatment. Community users who actively use the surveillance and reporting features as well as the medical care and logistical support features will be encouraged to comply with the protocols and measures to deal with the health disaster. An in-depth understanding of user compliance before designing a health EDMS is essential for governments and developers to increase the effectiveness of Health EDMS implementation.

## Appendix

Multimedia Appendix 1 Hazard classification by WHO [36]. WHO: World Health Organization.

Multimedia Appendix 2 PRISMA 2020 checklist. PRISMA: Preferred Reporting Items for Systematic Reviews and Meta-Analyses.

Multimedia Appendix 3 Studies included in the final review.

Multimedia Appendix 4 Factors affecting user compliance with Health EDMS. EDMS: emergency and disaster management system.

## Abbreviations

CTA: contact-tracing application
DMS: disaster management system
EDM: emergency and disaster management
EDMS: emergency and disaster management system
EMS: emergency management system
H7N9: avian influenza A
HBM: Health Belief Model
IS: information system
NGO: nongovernment organization
PADM: protective action decision model
PMT: protection motivation theory
PRISMA: Preferred Reporting Items for Systematic Reviews and Meta-Analyses
RACI: responsible, accountable, consulted, and informed
SARF: social amplification of risk framework
TAM2: extension of the technology acceptance model
TPB: theory of planned behavior
WHO: World Health Organization

## Data Availability

All data analyzed during this study are included in this published paper and its Multimedia Appendices.

## References

[1] Van de Walle B, Turoff M (2008). Decision support for emergency situations. Inf Syst E-Bus Manag.

[2] Waugh Jr WL (2015). Living with Hazards Dealing with Disasters: An Introduction to Emergency Management.

[3] (2017). Terminology on disaster risk reduction. United Nations Office for Disaster Risk Reduction.

[4] UN Secretary-General (2016). Report of the open-ended intergovernmental expert working group on indicators and terminology relating to disaster risk reduction, vol. 21184. United Nations Office for Disaster Risk Reduction.

[5] Albtoush R, Dobrescu R, Ionescou F (2011). A hierarchical model for emergency management systems. UPB Sci Bull Ser C Electr Eng.

[6] Rodrigues AS, Santos MA, Santos AD, Rocha F (2002). Dam-break flood emergency management system. Water Resour Manag.

[7] Wilder-Smith A, Osman S (2020). Public health emergencies of international concern: a historic overview. J Travel Med.

[8] Nikolic V, Savic S, Stankovic M (2007). Designing a multimedia platform for emergency management. Manag Environ Qual Int J.

[9] Sorensen JH, Mileti DS (2023). Decision-making uncertainties in emergency warning system organization. Int J Mass Emerg Disasters.

[10] Chen D, Liu Z, Wang L, Dou M, Chen J, Li H (2013). Natural disaster monitoring with wireless sensor networks: a case study of data-intensive applications upon low-cost scalable systems. Mobile Netw Appl.

[11] Erdelj M, Król M, Natalizio E (2017). Wireless sensor networks and multi-UAV systems for natural disaster management. Comput Netw.

[12] Kim SY, Yeniova AÖ (2022). Global, regional, and national incidence and mortality of COVID-19 in 237 countries and territories, January 2022: a systematic analysis for World Health Organization COVID-19 dashboard. Life Cycle.

[13] Jones L, Palumbo D, Brown D (2021). Coronavirus: how the pandemic has changed the world economy. BBC News.

[14] Kahn JP (2020). Digital Contact Tracing for Pandemic Respons: Ethics and Governance Guidance.

[15] Guo Y, An S, Comes T (2022). From warning messages to preparedness behavior: the role of risk perception and information interaction in the Covid-19 pandemic. Int J Disaster Risk Reduct.

[16] Alanzi T (2021). A review of mobile applications available in the App and Google Play Stores used during the COVID-19 outbreak. J Multidiscip Healthc.

[17] Blasimme A, Ferretti A, Vayena E (2021). Digital contact tracing against COVID-19 in Europe: current features and ongoing developments. Front Digit Health.

[18] Altay N, Green WG (2006). OR/MS research in disaster operations management. Eur J Oper Res.

[19] Rose DA, Murthy S, Brooks J, Bryant J (2017). The evolution of public health emergency management as a field of practice. Am J Public Health.

[20] Hu G, Rao K, Sun Z (2007). Identification of a detailed function list for public health emergency management using three qualitative methods. Chin Med J.

[21] Ming LC, Untong N, Aliudin NA, Osili N, Kifli N, Tan CS, Goh KW, Ng PW, Al-Worafi YM, Lee KS, Goh HP (2020). Mobile health apps on COVID-19 launched in the early days of the pandemic: Content analysis and review. JMIR Mhealth Uhealth.

[22] Abuhammad S, Khabour OF, Alzoubi KH (2020). COVID-19 contact-tracing technology: Acceptability and ethical issues of use. Patient Prefer Adherence.

[23] Ahmad M, Iram K, Jabeen G (2020). Perception-based influence factors of intention to adopt COVID-19 epidemic prevention in China. Environ Res.

[24] Altmann S, Milsom L, Zillessen H, Blasone R, Gerdon F, Bach R, Kreuter F, Nosenzo D, Toussaert S, Abeler J (2020). Acceptability of app-based contact tracing for COVID-19: cross-country survey study. JMIR Mhealth Uhealth.

[25] Blom AG, Wenz A, Cornesse C, Rettig T, Fikel M, Friedel S, Möhring K, Naumann E, Reifenscheid M, Krieger U (2021). Barriers to the large-scale adoption of a covid-19 contact tracing app in Germany: survey study. J Med Internet Res.

[26] Bradshaw EL, Ryan RM, Noetel M, Saeri AK, Slattery P, Grundy E, Calvo R (2020). Information safety assurances increase intentions to use COVID-19 contact tracing applications, regardless of autonomy-supportive or controlling message framing. Front Psychol.

[27] Dowthwaite L, Fischer J, Perez Vallejos E, Portillo V, Nichele E, Goulden M, McAuley D (2021). Public adoption of and trust in the nhs covid-19 contact tracing app in the united kingdom: quantitative online survey study. J Med Internet Res.

[28] Duan SX, Deng H (2021). Hybrid analysis for understanding contact tracing apps adoption. Ind Manag Data Syst.

[29] Garrett PM, White JP, Lewandowsky S, Kashima Y, Perfors A, Little DR, Geard N, Mitchell L, Tomko M, Dennis S (2021). The acceptability and uptake of smartphone tracking for COVID-19 in Australia. PLoS One.

[30] Grill E, Eitze S, De Bock F, Dragano N, Huebl L, Schmich P, Wieler LH, Betsch C (2021). Sociodemographic characteristics determine download and use of a corona contact tracing app in Germany-results of the COSMO surveys. PLoS One.

[31] Villius Zetterholm M, Lin Y, Jokela P (2021). Digital contact tracing applications during COVID-19: a scoping review about public acceptance. Informatics.

[32] Kahnbach L, Lehr D, Brandenburger J, Mallwitz T, Jent S, Hannibal S, Funk B, Janneck M (2021). Quality and adoption of COVID-19 tracing apps and recommendations for development: systematic interdisciplinary review of European apps. J Med Internet Res.

[33] Ribeiro-Navarrete S, Saura JR, Palacios-Marqués D (2021). Towards a new era of mass data collection: assessing pandemic surveillance technologies to preserve user privacy. Technol Forecast Soc Change.

[34] Kolasa K, Mazzi F, Leszczuk-Czubkowska E, Zrubka Z, Péntek M (2021). State of the art in adoption of contact tracing apps and recommendations regarding privacy protection and public health: systematic review. JMIR mHealth uHealth.

[35] Jandl C, Wagner M, Moser T, Schlund S (2021). Reasons and strategies for privacy features in tracking and tracing systems—a systematic literature review. Sensors (Basel).

[36] Golinelli D, Boetto E, Carullo G, Nuzzolese AG, Landini MP, Fantini MP (2020). Adoption of digital technologies in health care during the COVID-19 pandemic: systematic review of early scientific literature. J Med Internet Res.

[37] Bassi A, Arfin S, John O, Jha V (2020). An overview of mobile applications (apps) to support the coronavirus disease 2019 response in India. Indian J Med Res.

[38] Han W, Ada S, Sharman R, Rao HR (2015). Campus emergency notification systems: An examination of factors affecting compliance with alerts. MIS Q.

[39] Abdelhamid M, Howell P, Sharman R (2017). The impact of information and system quality on emergency notification systems adoption.

[40] O'Malley P, Rainford J, Thompson A (2009). Transparency during public health emergencies: from rhetoric to reality. Bull World Health Organ.

[41] Kumar M, Leroy G (2021). Factors affecting compliance with alerts in the context of healthcare-related emergencies. AIS Trans Replication Res.

[42] Page MJ, McKenzie JE, Bossuyt PM, Boutron I, Hoffmann TC, Mulrow CD, Shamseer L, Tetzlaff JM, Akl EA, Brennan SE, Chou R, Glanville J, Grimshaw JM, Hróbjartsson A, Lalu MM, Li T, Loder EW, Mayo-Wilson E, McDonald S, McGuinness LA, Stewart LA, Thomas J, Tricco AC, Welch VA, Whiting P, Moher D (2021). The PRISMA 2020 statement: an updated guideline for reporting systematic reviews. BMJ.

[43] (2015). Western Pacific Regional Framework for action for disaster risk management for health. World Health Organization.

[44] Blanchard BW (2008). Department of Homeland Security,.

[45] Emergency and disaster management. United Nations.

[46] (2013). Disaster response in Asia and the Pacific - a guide to international tools and services [EN/ID/JA/ZH]. UN Office for the Coordination of Humanitarian Affairs.

[47] (2002). World Health Organization.

[48] (2019). Health emergency and disaster risk management framework. World Health Organization.

[49] Duarte E, Rebelo F, Teles J, Noriega P (2012). What should I do? - A study about conflicting and ambiguous warning messages. Work.

[50] Wogalter MS, Woga MS (2019). Communication-human information processing (C-HIP) model. Forensic Human Factors & Ergonomics: Case Studies and Analyses.

[51] Lindell MK, Perry RW (2012). The protective action decision model: theoretical modifications and additional evidence. Risk Anal.

[52] Rahn M, Tomczyk S, Schopp N, Schmidt S (2021). Warning messages in crisis communication: risk appraisal and warning compliance in severe weather, violent acts, and the COVID-19 pandemic. Front Psychol.

[53] Sutton J, Spiro ES, Johnson B, Fitzhugh S, Gibson B, Butts CT (2013). Warning tweets: serial transmission of messages during the warning phase of a disaster event. Inf Commun Soc.

[54] Landau S (2008). Security and privacy landscape in emerging technologies. IEEE Secur Privacy Mag.

[55] Hannan A, Arshad S, Azam M, Loo J, Ahmed S, Majeed M, Shah S (2018). Disaster management system aided by named data network of things: architecture, design, and analysis. Sensors (Basel).

[56] Currion P, Silva CD, Van de Walle B (2007). Open source software for disaster management. Commun ACM.

[57] Amailef K, Lu J (2013). Ontology-supported case-based reasoning approach for intelligent m-government emergency response services. Decis Support Syst.

[58] Edwards DA, Cuthbertson H, Peterson D (2020). Developing and testing an emergency notification system for a county emergency management agency. J Homel Secur Emerg Manag.

[59] Malizia A, Acuna P, Onorati T, Diaz P, Aedo I (2009). CAP-ONES: an emergency notification system for all. Int J Emerg Manag.

[60] Ouyang L, Yuan Y, Cao Y, Wang F-Y (2021). A novel framework of collaborative early warning for COVID-19 based on blockchain and smart contracts. Inf Sci (N Y).

[61] Bonaretti D, Fischer-Preßler D (2021). The problem with SMS campus warning systems: an evaluation based on recipients’ spatial awareness. Int J Disaster Risk Reduct.

[62] Sharma S, Singh G, Sharma R, Jones P, Kraus S, Dwivedi YK (2020). Digital health innovation: exploring adoption of COVID-19 digital contact tracing apps. IEEE Trans Eng Manag.

[63] Tomczyk S, Barth S, Schmidt S, Muehlan H (2021). Utilizing health behavior change and technology acceptance models to predict the adoption of COVID-19 contact tracing apps: cross-sectional survey study. J Med Internet Res.

[64] Ristvej J, Zagorecki A (2011). Information aystems for crisis management - current applications and future directions. Komunikácie.

[65] Mitraka Z, Siachalou S, Doxani G, Patias P (2020). Decision support on monitoring and disaster management in agriculture with Copernicus sentinel applications. Sustainability.

[66] Damalas A, Mettas C, Evagorou E, Giannecchini S, Iasio C, Papadopoulos M, Konstantinou A, Hadjimitsis D (2018). Development and implementation of a DECATASTROPHIZE platform and tool for the management of disasters or multiple hazards. Int J Disaster Risk Reduct.

[67] Liu X, Lim S (2016). Integration of spatial analysis and an agent-based model into evacuation management for shelter assignment and routing. J Spat Sci.

[68] He Y, Zhang D, Fang Y (2017). Development of a mobile post-disaster management system using free and open source technologies. Int J Disaster Risk Reduct.

[69] Ding Y, Fan Y, Du Z, Zhu Q, Wang W, Liu S, Lin H (2014). An integrated geospatial information service system for disaster management in China. Int J Digit Earth.

[70] Thomas R, Michaleff ZA, Greenwood H, Abukmail E, Glasziou P (2020). Concerns and misconceptions about the Australian government's COVIDsafe app: cross-sectional survey study. JMIR Public Health Surveill.

[71] Mbunge E (2020). Integrating emerging technologies into COVID-19 contact tracing: opportunities, challenges and pitfalls. Diabetes Metab Syndr.

[72] (2020). Which countries are deploying coronavirus tracing apps?. Statista.

[73] (2022). WHO coronavirus (COVID-19) dashboard. World Health Organization.

[74] Étienne J (2010). Compliance theories: a literature review. Rev Fr Sci Polit.

[75] Etzioni A (1961). An analytical classification coercive and utilitarian organizations. A Comparative Analysis of Complex Organizations.

[76] Kyngäs H, Duffy ME, Kroll T (2000). Conceptual analysis of compliance. J Clin Nurs.

[77] Kuppusamy P, Samy GN, Maarop N, Magalingam P, Kamaruddin N, Shanmugam B, Perumal S (2020). Systematic literature review of information security compliance behaviour theories. J Phys: Conf Ser.

[78] Zhou J, Fang Y, Grover V (2022). Managing collective enterprise information systems compliance: a social and performance management context perspective. MIS Q.

[79] Sorensen JH (2000). Hazard warning systems: review of 20 years of progress. Nat Hazards Rev.

[80] Kitchenham B (2004). Procedures for performing systematic reviews. Tech Rep.

[81] Kitchenham B, Charters S (2007). Guidelines for performing systematic literature reviews in Software Engineering version 2.3. Engineering.

[82] Meyer J (2004). Conceptual issues in the study of dynamic hazard warnings. Hum Factors.

[83] Laughery KR (2006). Safety communications: warnings. Appl Ergon.

[84] Freberg K (2012). Intention to comply with crisis messages communicated via social media. Public Relat Rev.

[85] Albrecht R, Jarecki JB, Meier DS, Rieskamp J (2021). Risk preferences and risk perception affect the acceptance of digital contact tracing. Humanit Soc Sci Commun.

[86] Panchal M, Singh S, Rodriguez-Villegas E (2021). Analysis of the factors affecting the adoption and compliance of the NHS COVID-19 mobile application: a national cross-sectional survey in England. BMJ Open.

[87] Wang F, Wei J, Shi X (2018). Compliance with recommended protective actions during an H7N9 emergency: a risk perception perspective. Disasters.

[88] Guillon M, Kergall P (2020). Attitudes and opinions on quarantine and support for a contact-tracing application in France during the COVID-19 outbreak. Public Health.

[89] Lee M, You M (2021). Effects of COVID-19 emergency alert text messages on practicing preventive behaviors: cross-sectional web-based survey in South Korea. J Med Internet Res.

[90] Rogers CJ, Forster M, Bahr K, Benjamin SM (2021). A cross-sectional study using health behavior theory to predict rapid compliance with campus emergency notifications among college students. Disaster Med Public Health Prep.

[91] Fricke P, Etzioni A (1976). A comparative analysis of complex organizations. Polit Sci Q.

[92] Rosenstock IM (1974). The Health Belief Model and preventive health behavior. Health Educ Monogr.

[93] Abraham C, Sheeran P (2014). The health belief model. Cambridge Handbook of Psychology, Health and Medicine, 2nd edition.

[94] Rogers R, Cacioppo J, Petty R, Cacioppo JT, Petty R (1983). Cognitive and physiological processes in fear appeals and attitude change: a revised theory of protection motivation. Basic Social Psycophysiological Research.

[95] Plotnikoff RC, Trinh L (2010). Protection motivation theory: is this a worthwhile theory for physical activity promotion?. Exerc Sport Sci Rev.

[96] Kasperson RE, Renn O, Slovic P, Brown HS, Emel J, Goble R, Kasperson JX, Ratick S (1988). The social amplification of risk: a conceptual framework. Risk Anal.

[97] Venkatesh V, Davis FD (2000). A theoretical extension of the technology acceptance model: four longitudinal field studies. Manag Sci.

[98] Ajzen I (1991). The theory of planned behavior. Org Behav Hum Decis Process.

