# Supplementary material for: User Compliance With the Health Emergency and Disaster Management System: Systematic Literature Review
Source: J Med Internet Res. 2023 May 5;25:e41168. doi: 10.2196/41168 (PMC10199396; doi:10.2196/41168)
Supplement: Multimedia Appendix 1 [file jmir_v25i1e41168_app1.docx]

| Natural hazards | Geophysical | earthquake, tsunami, mass movement, liquefaction, volcanic activity |
| --- | --- | --- |
|  | Hydro-Meteorological | hydrological: flood, mass movement, wave action  meteorological: storm, extreme temperature, fog  climatological: drought, wildfire, glacial lake outburst |
|  | Biological | airborne diseases, waterborne diseases, vector-borne diseases, foodborne outbreaks, insect infestation, animal diseases, plant diseases, aeroallergens, antimicrobial-resistant microorganisms, animal-human contact - venomous animals |
|  | Extraterrestrial | impact, space weather |
| Human-Induced hazards | Technological | industrial hazards, structural collapse, occupational hazards, transportation, explosions, fire, air pollution, infrastructure disruption, cybersecurity, hazardous materials in air, soil, water, food contamination |
|  | Societal | acts of violence, armed conflicts, civil unrest, stampedes, terrorism, financial crises |
| Environmental hazards | Environmental Degradation | erosion, deforestation, salinization, sea level rise, desertification, wetland loss/ degradation, glacier retreat/ melting, sand encroachment |
