# Supplementary material for: User Compliance With the Health Emergency and Disaster Management System: Systematic Literature Review
Source: J Med Internet Res. 2023 May 5;25:e41168. doi: 10.2196/41168 (PMC10199396; doi:10.2196/41168)
Supplement: Multimedia Appendix 3 [file jmir_v25i1e41168_app3.docx]

| **No** | **Reference** | **Publication Outlet** | **Study Location** | **Theory** | **Factors** | **Method** |
| --- | --- | --- | --- | --- | --- | --- |
| 1 | [85] | Humanities and Social Sciences Communications | Switzerland | - | Risk perception, risk seeking preference | Survey |
| 2 | [27] | Journal of Medical Internet Research | UK | TAM | Subjective norm, job relevance, output quality, result demonstrability, trust, perceived usefulness, perceived ease of use, experience, voluntariness | Survey |
| 3 | [84] | Public Relations Review | USA | - | Warning messages characteristics | Experiment |
| 4 | [88] | Public Health | France | HBM | Perceived susceptibility, perceived severity, perceived benefit, perceived barriers, cues to action, perceived threat, risk seeking preference | Survey |
| 5 | [15] | International Journal of Disaster Risk Reduction | China | PADM and SARF | Protective action perception, warning message characteristics, stakeholder perception, information interaction | Survey |
| 6 | [38] | MIS Quarterly | USA | Etzioni's compliance theory | Subjective norm, perceived safety threat, perceived financial threat, information quality trust, experience | Experiment and Focus Group Discussion |
| 7 | [41] | AIS Transactions on Replication Research | USA | Etzioni's compliance theory | Subjective norm, information quality trust | Experiment |
| 8 | [83] | Applied Ergonomics | USA | - | Perceived barriers, knowledge, subjective norm | Conceptual paper |
| 9 | [82] | Human Factors | Israel | - | Risk perception, warning message characteristics, perceived usefulness, trust in system, usability | Conceptual paper |
| 10 | [86] | BMJ Open | UK | - | knowledge, warning message characteristics, usability, security and privacy concerns | Survey |
| 11 | [52] | Frontiers in psychology | Germany | PADM | *Unsignificant factors* | Experiment |
| 12 | [90] | Disaster Medicine and Public Health Preparedness | USA | TPB and PMT | Attitude, subjective norm, perceived behavioral control | Experiment |
| 13 | [87] | Disasters | China | HBM and PADM | Stakeholder perception, protective action perception, risk perception | Survey |
| 14 | [89] | Journal of Medical Internet Research | South Korea | PMT | Reading text message, response efficacy | Survey |
