# Supplementary material for: User Compliance With the Health Emergency and Disaster Management System: Systematic Literature Review
Source: J Med Internet Res. 2023 May 5;25:e41168. doi: 10.2196/41168 (PMC10199396; doi:10.2196/41168)
Supplement: Multimedia Appendix 4 [file jmir_v25i1e41168_app4.docx]

| **No** | **Factor** | **Definition** | **Theory** | **References** | **CTA compliance**  **studies** |
| --- | --- | --- | --- | --- | --- |
| **Individual Factors** | | | | | |
| 1 | Perceived threat or perceived safety threat | Beliefs about the likelihood and consequences of potential threats | PMT, PADM, Etzioni’s | [38], [88] | TousAntiCovid (France) [88] |
| 2 | Risk perception, perceived susceptibility or vulnerability | Beliefs about the likelihood toward experiencing a potential threat | HBM, PMT | [82], [85], [87], [88] | SwissCovid (Switzerland) [85], TousAntiCovid (France) [88] |
| 3 | Response efficacy or protective action perception | Belief that taking protective action is effective in reducing the risk | PMT, PADM | [15], [87], [89] | N/A (China) [15] |
| 4 | Perceived barriers | Assessment of the obstacles to behavior change | HBM | [83], [88] | TousAntiCovid (France) [88] |
| 5 | Perceived severity | Beliefs about the seriousness of the consequences of the condition | HBM, PMT | [88] | TousAntiCovid (France) [88] |
| 6 | Perceived financial threat | Perception of potential financial loss due to security-related incidents | Etzioni's | [38] | - |
| 7 | Knowledge | Familiarity with the system and emergency plan | - | [83], [86] | NHS COVID-19 (UK) [86] |
| 8 | Attitude | The extent of a favorable or unfavorable appraisal of a behavior | TPB | [90] | - |
| 9 | Perceived behavioral control | Individual's confidence to execute a given behavior | TPB | [90] | - |
| 10 | Voluntariness | Willingness to do something voluntarily | TAM2 | [27] | NHS COVID-19 (UK) [27] |
| 11 | Risk seeking preference | Level of aversion or tolerance regarding certain types of risk | - | [85], [88] | SwissCovid (Switzerland) [85], TousAntiCovid (France) [88] |
| 12 | Experience | Direct experience in a disaster conditions | TAM2 | [27], [38] | NHS COVID-19 (UK) [27] |
| 13 | Perceived Benefit | The efficiency of protective action to protect the general population and trust in government to handle the health crisis | HBM | [88] | TousAntiCovid (France) [88] |
| 14 | Reading text message | User's activity to read and obtain information via emergency alert messages |  | [89] |  |
| **Technological Factors** | | | | | |
| 1 | Warning messages characteristics | Attributes of the warning delivered, including the message's source, channel, and content | PADM | [15], [82]–[84], [86] | NHS COVID-19 (UK) [86], N/A (China) [15] |
| 2 | Perceived usefulness | Assessment that using a system will improve performance or provide benefits | TAM2, HBM | [27], [82] | NHS COVID-19 (UK) [27] |
| 3 | Information quality trust | Beliefs about the reliability, credibility, and accuracy of information | - | [38], [41] | - |
| 4 | Trust in the system | Level of trust in distinct aspects of the system | - | [27], [82] | NHS COVID-19 (UK) [27] |
| 5 | Usability | Users' views of their ability to use and navigate the system | - | [82], [86] | NHS COVID-19 (UK) [86] |
| 6 | Perceived ease of use | How users expect the system to be effort-free | TAM2 | [27] | NHS COVID-19 (UK) [27] |
| 7 | Job relevance | The degree to which the system is applicable to a person's job | TAM2 | [27] | NHS COVID-19 (UK) [27] |
| 8 | Output quality | Tasks that can be performed by the system and the degree of relevance to the job | TAM2 | [27] | NHS COVID-19 (UK) [27] |
| 9 | Result demonstrability | The tangibility of the results of using the system | TAM2 | [27] | NHS COVID-19 (UK) [27] |
| 10 | Security and privacy concern | User views on system privacy and security | - | [86] | NHS COVID-19 (UK) [86] |
| **Social Factors** | | | | | |
| 1 | Subjective norm or social influence | Perceived social and peer pressures to perform or not perform a particular behavior | TAM2, TPB, Etzioni’s | [27], [38], [41], [83], [90] | NHS COVID-19 (UK) [27] |
| 2 | Stakeholder perception | Stakeholder cognitive evaluation of hazards | PADM | [15], [87] | N/A (China) [15] |
| 3 | Cues to action | Internal or external triggers to perform an action | HBM | [88] | TousAntiCovid (France) [88] |
| 4 | Information interaction | Discussions and exchange of information with others | SARF | [15] | N/A (China) [15] |
